# Building and Expanding a Multidisciplinary Pediatric Epilepsy Surgery Program: Perspectives From a Children’s Hospital in an Underserved Area

**DOI:** 10.7759/cureus.90174

**Published:** 2025-08-15

**Authors:** Lisa B Shields, Ian S Mutchnick, Shannon D Davis, Brandy Duvall-Howard, Annette A Stambaugh, Jennifer Kenney, Bronson L Howard, Kathy Lamb, Vinay Puri, Cemal Karakas

**Affiliations:** 1 Neurological Surgery, Norton Neuroscience Institute, Norton Healthcare, Louisville, USA; 2 Neurosurgery, Norton Children's Hospital, Louisville, USA; 3 Neurology, Norton Neuroscience Institute, Norton Healthcare, Louisville, USA; 4 Neurology, Norton Children's Neuroscience Institute and Children's Hospital, Norton Healthcare, Louisville, USA; 5 Pediatric Neurology, University of Louisville School of Medicine, Louisville, USA

**Keywords:** epilepsy, pediatric, pediatric neurology, pediatric neurosurgery, pediatric surgery, seizures

## Abstract

Children with epilepsy commonly develop drug-resistant epilepsy, which often necessitates surgical intervention. Timely access to surgical intervention is critical for optimizing outcomes in these children. However, the significant undersupply of qualified epilepsy centers ensures that there remains a pressing need to expand and support pediatric epilepsy centers. This perspective article presents the development and expansion of our Pediatric Epilepsy Surgery Program in Kentucky. Overcoming initial obstacles such as limited infrastructure, staffing, and resources allowed us to implement strategic planning, acquire advanced neurosurgical and neurodiagnostic equipment, increase staff training, and establish subspecialty pediatric epilepsy clinics. Our program boasts several innovative technologies, including Visualase laser ablation, the Surgical Theater 3D visualization platform, responsive neurostimulation, deep brain stimulation, and robotic-assisted stereoelectroencephalography. Since its inception, our program has seen a substantial increase in surgical volume and has attained national recognition for its comprehensive and cutting-edge approach to pediatric epilepsy care. This article aims to describe the strategic development, infrastructure planning, staffing, clinical implementation, and outcomes of a pediatric epilepsy surgery program in a low-resource setting, offering a model for replication. This paper outlines the key steps involved in developing and expanding our Pediatric Epilepsy Surgery Program, which may serve as a valuable model for other institutions seeking to establish or grow their own pediatric epilepsy surgery programs, particularly in regions with limited access to comprehensive epilepsy care.

## Introduction

According to the 2022 National Survey of Children’s Health, approximately 456,000 children suffer from epilepsy in the United States (US) [1]. Epilepsy is the most common childhood brain disorder in the US [2]. Between 30% and 40% of children with epilepsy have refractory seizures, which may negatively impact child development, including education, performance, and social development [3]. Although pediatric epilepsy shares several characteristics with adult epilepsy, the developing brain in the child and adolescent is associated with different epilepsy etiologies, syndromes, comorbidities, choice of appropriate investigations, surgical interventions, and outcomes [4,5]. The long-term potential complications of epilepsy and accompanying treatments remain to be elucidated. Additionally, newly available epilepsy therapies approved for adults by the US Food and Drug Administration (FDA) may not be concurrently approved in the pediatric population, and pediatric patients may need to be sedated for procedures, unlike adults [4]. It has been reported that earlier surgery may be more likely to lead to seizure freedom with improved cognitive and psychosocial outcomes [6]. Surgery may be destructive (removing or disconnecting the seizure focus), or neuromodulatory (responsive neurostimulation (RNS), deep brain stimulation (DBS), or vagal nerve stimulation (VNS)) [7]. Epilepsy surgery requires sophisticated equipment, knowledge, and a multidisciplinary approach that are not available in many centers.

Due to the unique features inherent in pediatric epilepsy, it is imperative for pediatric epileptic patients to be closely monitored by a multidisciplinary team of providers who are knowledgeable about the myriad opportunities for medical management and surgical intervention for epilepsy targeting the pediatric population. Our state, Kentucky, has a relatively low socioeconomic status compared to other states in the US. It has one of the highest poverty rates and lowest median household incomes. In 2023, Kentucky had the sixth-highest poverty rate (16.4%) and fifth-highest child poverty rate (21%) in the US [8]. Kentucky also has more residents with epilepsy than any other state [9]. Kentucky faces numerous challenges with respect to pediatric epilepsy care, including prolonged delays in diagnosis, limited treatment options, a lack of available modern resources to treat epilepsy, and the need to travel long distances to receive advanced epilepsy care [9]. Taking the socioeconomic and geographic disparities into consideration, our children’s hospital sought to create a comprehensive pediatric epilepsy surgery program.

Our pediatric epilepsy center uses innovative technology such as Visualase (aids in performing magnetic resonance imaging (MRI)-guided laser ablation surgery) and the Surgical Theater system (virtual reality technology that converts multiple two-dimenional tests into an integrated three-dimensional interactive model) to treat patients with epilepsy and deep brain tumors who previously would not have been candidates for surgery. The team of neurosurgeons and neurologists at our center provides a comprehensive, multidisciplinary approach to treating epilepsy in infants, children, and adolescents by creating a customized treatment plan. This article aims to describe the strategic development, infrastructure planning, staffing, clinical implementation, and outcomes of a pediatric epilepsy surgery program in a low-resource setting, offering a model for replication. Herein, we present the development and expansion of our Pediatric Epilepsy Surgery Program, including the (1) initial goals of our program, (2) diagnostic and surgical equipment for pediatric epilepsy, (3) expansion and education of staff, (4) surgical techniques and neuromodulation, (5) neurodiagnostic advancements, (6) program outcomes, and (7) specialized pediatric epilepsy clinics. We also highlight the (8) research accomplishments and (9) future goals of our center. Epilepsy surgery centers in low socioeconomic countries are also described.

## Materials and methods

Our Pediatric Epilepsy Surgery Program was established at Norton Children’s Hospital in Louisville, Kentucky in January 2020. We initially performed a comprehensive needs assessment by analyzing the regional healthcare data to determine the unmet needs in pediatric epilepsy care. The goals of our Pediatric Epilepsy Surgery Program are presented in Table 1. The primary goals included developing a diagnostic and surgical infrastructure, specialized epilepsy clinics, and educational and research opportunities. Providing patient care in a timely manner and encouraging collaboration among the members of the multidisciplinary pediatric epilepsy team were also promoted.

**Table 1:** Goals of Our Pediatric Epilepsy Surgery Program

Goals	Specific Details of Each Goal
Establish an advanced epilepsy diagnostic and surgical infrastructure	Advanced neuroimaging capabilities to accurately determine seizure locations (functional MRI and robotic-assisted intracranial EEG implantation) and minimally invasive surgical technologies (laser ablation)
Foster multidisciplinary collaboration	Integrate neurology, neurosurgery, neuropsychology, neuroradiology, nursing, and rehabilitation teams to provide comprehensive, patient-centered pediatric epilepsy care
Create specialized epilepsy clinics	Services for specific epilepsy subtypes (refractory epilepsy, neuromodulation, tuberous sclerosis complex, epileptic spasms, ketogenic diet, adolescent with epilepsy)
Provide care in a timely manner	Rapid identification and comprehensive management of surgical candidates
Establish educational opportunities	Fellowship and educational programs to train pediatric epilepsy specialists and provide ongoing professional development for existing staff
Promote clinical research and innovation	Develop clinical and translational research contexts, encourage program improvement through evidence-based practices, and collaborate on projects with national epilepsy research groups

**Figure 1:**
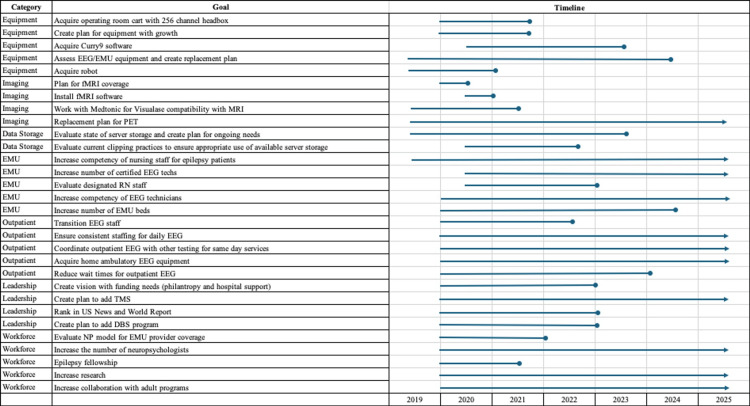
Strategic Decision-Making Process at Our Pediatric Epilepsy Surgery Program EMG: electromyography; EEG: electroencephalogram; RN: registered nurse; EMU: epilepsy monitoring unit; fMRI: functional magnetic resonance imaging; TMS: transcranial magnetic stimulation; DBS: deep brain stimulation; NP: nurse practitioner Image credits: Norton Children’s Hospital, Pediatric Epilepsy Team

A multidisciplinary pediatric epilepsy surgery committee consisted of pediatric epileptologists and neurosurgeons, neuropsychologists, radiologists, nurse practitioners, nurse navigators, electroencephalography (EEG) technologists, and hospital administrators. This committee composed a strategic five-year development plan with goals, timelines, and resource needs. Figure 1 depicts the strategic decision-making process at our Pediatric Epilepsy Surgery Program, including the prioritization of investments, timeline management, and function mechanism (philanthropy and hospital support). All of the goals in this plan have been met.

Clinical pathway development

**Figure 2:**
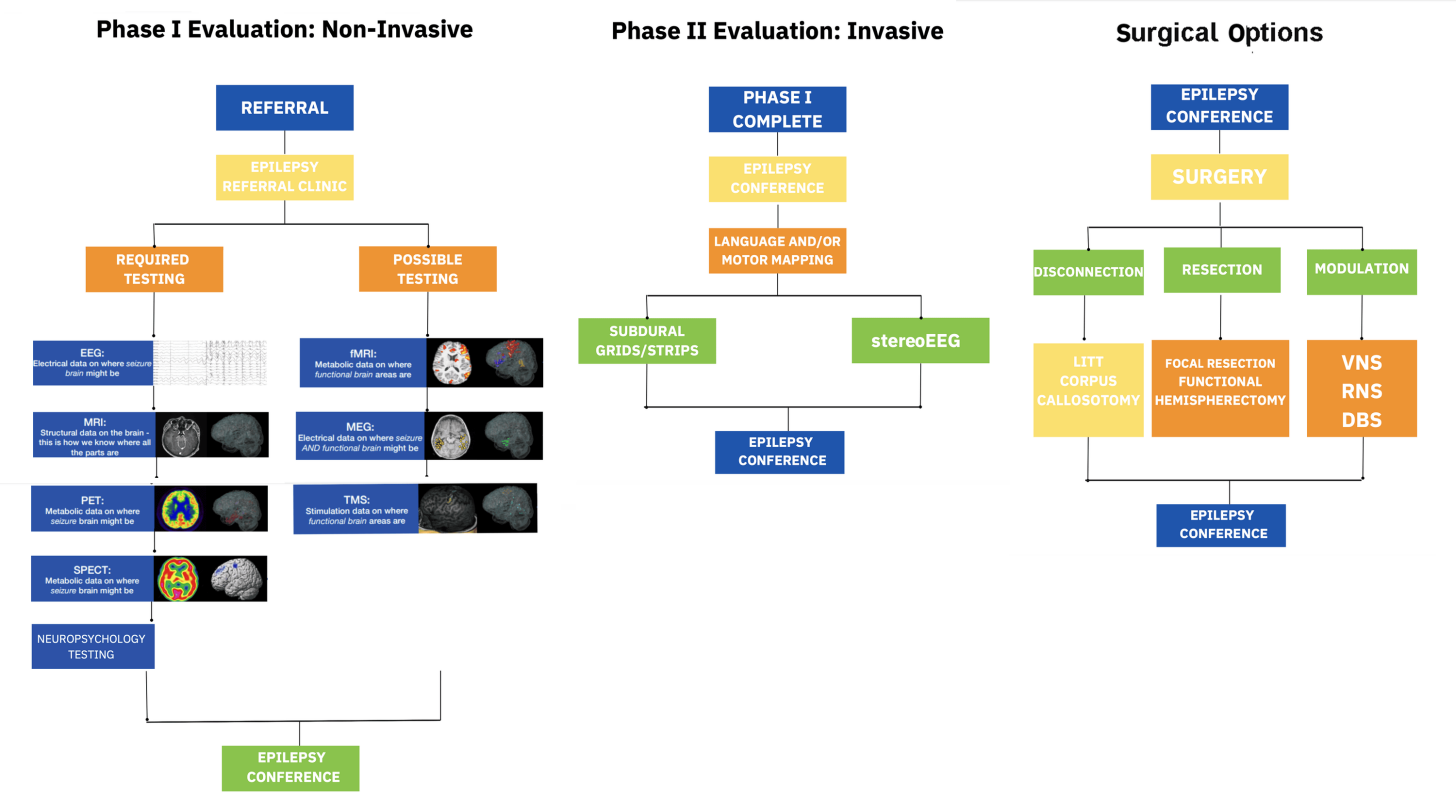
Pathway Algorithm at Our Pediatric Epilepsy Surgery Program Algorithm highlighting the pathways from patient referral to our Pediatric Epilepsy Surgery Program to Phase I evaluation, Phase II assessment, and to surgical intervention for epilepsy. EMU: epilepsy monitoring unit; fMRI: functional magnetic resonance imaging; MEG: magnetoencephalography; MRI: magnetic resonance imaging; PET: positron emission tomography; SPECT: single photon emission computed tomography; EEG: electroencephalogram; TMS: transcranial magnetic stimulation; LITT: laser interstitial thermal therapy; VNS: vagus nerve stimulation; RNS: responsive neurostimulation; DBS: deep brain stimulation Image credits: Norton Children’s Hospital, Pediatric Epilepsy Team

Figure 2 highlights an algorithm encompassing the pathways from patient referral to our clinic to Phase I evaluation (3T-MRI, positron emission tomography (PET), single positron emission computed tomography (SPECT), video-EEG, functional MRI (fMRI), magnetoencephalography (MEG), and neuropsychology testing), Phase II assessment (stereo-EEG, subdural grid EEG and cortical language and/or motor mapping), and potentially surgical intervention for epilepsy. Clinical pathways streamlined the evaluation of pediatric patients with epilepsy from the initial consultation to postoperative follow-up. Patient navigators facilitate patient and family communication, scheduling, and education throughout the surgical process.

All patient outcomes were measured by success criteria, patient satisfaction, and seizure control rates. All outcomes were measured by postoperative EEG data one year postoperatively. Additionally, patients were evaluated by an epileptologist every three months postoperatively, at which time patient measures were reported. Adult patients reported whether they were able to attend college or go to work. School-age patients obtained reports from teachers. All patients underwent neuropsychology testing one year postoperatively.

## Results

Resource acquisition and neurodiagnostic unit advancements

Through multidisciplinary efforts, our center was able to acquire myriad innovative diagnostic and surgical equipment required for epilepsy surgery, including 3T-MRI, PET, SPECT, Wada study, portable MRI, stereo-EEG, subdural grid EEG, neonatal EEGs, intraoperative electrocorticography (ECoG), and electromyography (EMG) (Table 2). Our Epilepsy Monitoring Unit (EMU) was a $3 million investment for our hospital, $280,000 of which was funded by the WHAS Crusade for Children [10].

**Table 2:** Acquisition of Diagnostic and Surgical Equipment for Epilepsy Surgery EEG: electroencephalogram; MRI: magnetic resonance imaging; CT: computed tomography; sEEG: stereoelectroencephalography

Equipment	Specific Equipment’s Use
256-Channel EEG headbox Natus® (Middleton, WI)	Improves spatial resolution in scalp and intracranial EEG recordings; more precise identification and delineation of seizure foci
Curry 9 Software systems Compumedics NeuroScan^TM^ (Charlotte, NC)	Provides advanced 3D EEG source localization and neuroimaging co-registration; enhances presurgical evaluation accuracy and surgical outcomes
Outpatient EEG equipment Natus® (Middleton, WI) and Nihon Kohden (Irvine, CA)	Permits long-duration EEG recordings at home, capturing seizures not seen during brief hospital stays; improves the diagnostic yield of surgical candidacy evaluations
Visualase^TM^ MRI-Compatible Laser Ablation Technology Medronic (Minneapolis, MN)	Minimally invasive surgical option reduces surgical morbidity, hospital stays, and postoperative recovery time, which increases patient and family satisfaction
Surgical Theater Surgical Theater (Beachwood, OH)	Virtual reality technology that converts two-dimensional radiographic datasets (such as MRI and CT) to three-dimensional interactive models; used for sEEG planning; facilitates epilepsy decision-making for diagnosis and treatment, improves surgical navigation, and enables complex epilepsy surgeries
Robotic sEEG System Medronic (Minneapolis, MN)	Improves the accuracy, efficiency, and safety of electrode placement
Functional MRI Siemens Healthineers (Washington, DC)	Installation of software for advanced presurgical functional cortical mapping; allows precise surgical planning and minimizes postoperative functional deficits
Server and data management	Extensive server storage enhancements permit secure and efficient handling of large volumes of imaging and EEG data, which enhances streamlining of multidisciplinary case reviews and patient management

Neurodiagnostic advancements were made to the EEG infrastructure, outpatient EEG growth, EMU expansion, and fMRI integration (Table 3). As of May 2025, our center has expanded to eight EMU beds, each with two high-resolution cameras, and two satellite EMU beds. We also have 10 continuous EEG machines, which perform daily inpatient EEGs as well as 10-15 outpatient EEGs.

**Table 3:** Neurodiagnostic Unit Advancements at Our Pediatric Epilepsy Surgery Program EEG: electroencephalogram; EMU: epilepsy monitoring unit; fMRI: functional magnetic resonance imaging

Neurodiagnostic Unit Advancements	Specific Details
EEG infrastructure	Transitioned from a traditional 24-channel EEG system to a modern 256-channel EEG acquisition system capable of simultaneous intracranial and scalp recordings; the Curry 9 software permitted advanced EEG source localization, facilitating highly accurate presurgical evaluations
Outpatient EEG growth	The outpatient EEG program grew dramatically each year since its inception; increased diagnostic accuracy and captured seizures previously undocumented
EMU expansion	Expanded from an initial four beds to eight dedicated beds and weekend EMU admissions with continuous video EEG monitoring, which significantly reduced wait times from six months to six weeks; increased the identification and surgical candidacy rates of pediatric patients with refractory epilepsy
Functional MRI integration	Integrated fMRI to perform precise presurgical language and motor mapping to better preserve neurological functions during surgery

Specialized staffing development and training

Our Pediatric Epilepsy Surgery Program expanded specialized epilepsy staffing who received training specific to their position. Table 4 depicts the staff positions at our Pediatric Epilepsy Surgery Program as of May 2025. Our center follows the American Clinical Neurophysiology Society’s (ACNS) guidelines for monitoring, staffing, and clinical practice in the EMU [11].

**Table 4:** Specialized Staffing Development and Training EEG: electroencephalogram; EMU: epilepsy monitoring unit; APRN: advanced practice registered nurse; PA: physician assistant; RN: registered nurse; R. EEG T: registered EEG technologists

Staff Positions	Number of Each Staff Position
EEG readers	9 (7 epilepsy fellowship-trained pediatric epileptologists and 2 additional general pediatric neurologists who are EEG readers)
EMU/EEG nursing staff and technologists	27
EMU APRNs	2
EMU PA	1
Outpatient epilepsy APRNs	3
Outpatient epilepsy clinic APRNs	3
Neuropsychologist	1
Pediatric neurosurgeons	3 (one with a special interest in epilepsy surgery)
Radiologists	2
Epilepsy nurse navigators	2
Epilepsy RN clinician/educator	1
Full-time R. EEG T	4

Our Pediatric Epilepsy Surgery Program created multidisciplinary epilepsy surgery weekly conferences attended by epileptologists, neurosurgeons, radiologists, neuropsychologists, and nurses. This group discusses complex refractory epilepsy cases, consensus-driven surgical planning, and postoperative patient follow-up. Extensive staff training, continuous quality improvement, and recurring outcome monitoring and analysis were implemented.

Pediatric Epilepsy Surgery Program surgical advancements

Several surgical advancements have been developed at our Pediatric Epilepsy Surgery Program, including the introduction and optimization of laser ablation, minimally invasive techniques, and advanced neuromodulation (RNS and DBS). Stereo-EEG and robot-assisted surgeries have also been implemented. A total of 169 surgeries have been performed from our program’s inception in January 2020 to May 2025 (Table 5). The number of these cases (excluding VNS) increased over the duration of our program: 2020 (6 cases), 2021 (14 cases), 2022 (19 cases), 2023 (16 cases), 2024 (16 cases), and until May 2025 (7 cases). The postoperative seizure outcomes at our Pediatric Epilepsy Surgery Program are presented in Table 6.

**Table 5:** Pediatric Epilepsy Surgery Program Surgical Advancements VNS: vagal nerve stimulation; sEEG: stereoelectroencephalography; RNS: responsive neurostimulation; LITT: laser interstitial thermal therapy; DBS: deep brain stimulation

Types of Pediatric Epilepsy Surgeries	Number of Each Type of Surgery (n=169)
VNS	87
sEEG	33
Resection	9
Corpus callosotomy	9
RNS	10
LITT	10
Hemispherectomy	9
DBS	2

**Table 6:** Postoperative Seizure Outcomes at Our Pediatric Epilepsy Surgery Program RNS: responsive neurostimulation; LITT: laser interstitial thermal therapy; DBS: deep brain stimulation; SUDEP: sudden unexpected death in epilepsy

Pediatric Epilepsy Surgery	Postoperative Seizure Outcomes
RNS	Total: 10 surgeries
	3 cases: >50% improvement
	3 cases: 90% seizure freedom
	2 cases: performed too recently to determine outcomes
	1 case: increase in seizure frequency
	1 case: postoperative incision site infection, RNS device explanted
LITT	Total: 10 surgeries
	6 cases: 100% seizure freedom
	1 case: 90% seizure freedom
	1 case: 50% seizure frequency
	1 case: same seizure frequency as preoperatively
	1 case: lost to follow-up
	Two of these patients are now adults and are able to work, and 2 others went to college; all patients with seizure freedom reported a better quality of life
Resections	Total: 9 surgeries
	7 cases: 100% seizure freedom
	1 case: 90% seizure freedom
	1 case: lost to follow-up
	Of the 8 surgeries with follow-up, 90-100% had seizure freedom, all reported a better quality of life, better grades in school, independence to work jobs, less anxiety in social situations, and better affect from seizure freedom
Hemispherectomies	Total: 9 surgeries
	6 cases: 100% seizure freedom
	1 case: 90% seizure freedom
	1 case: 70% seizure freedom
	1 case: performed too recently to determine outcomes
	4 of these patients reported a better quality of life; all postoperative EEG reports showed improvement or showed no seizure frequency.
Corpus callosotomy	Total: 9 surgeries
	8 cases: 50% seizure freedom
	1 case: patient died 3 years later, likely due to SUDEP
DBS	Total: 2 surgeries
	1 case: >50% seizure freedom
	1 case: performed too recently to determine outcomes

Specialized clinical services

Our program has established numerous specialized epilepsy outpatient clinics. In addition to the specialized clinics, myriad multidisciplinary clinics are also available (Table 7).

**Table 7:** Specialized Multidisciplinary Clinical Services RNS: responsive neurostimulation; DBS: deep brain stimulation

Multidisciplinary Clinical Services
Ketogenic diet
Tuberous sclerosis
Neuromodulation (RNS/DBS)
Infantile spasms
New onset seizures
Adolescent women with epilepsy
Functional neurological disorders
Autism
Neurogenetics

Epilepsy fellowship

The epilepsy/EEG fellowship program was established in 2022, with the first epilepsy fellow admitted in 2023. As of June 2025, two EEG/epilepsy fellows have graduated from this program. We established a one-year Accreditation Council for Graduate Medical Education (ACGME)-accredited epilepsy fellowship and a one-year non-ACGME-accredited EEG fellowship. Table 8 highlights the components of the epilepsy fellowship.

**Table 8:** Components of the Epilepsy Fellowship vEEG: videoelectroencephalography; sEEG: stereoelectroencephalography; EEG: electroencephalography; ECoG: electrocorticography; RNS: responsive neurostimulation; DBS: deep brain stimulation; AES: American Epilepsy Society; AAN: American Academy of Neurology

Epilepsy Fellowship Components
Core rotations: vEEG, sEEG, subdural EEG, epilepsy surgery and genetics, neuromodulation (RNS/DBS) treatment, brain mapping, neurophysiology, and ECoG
Weekly continuity clinic
Educational conferences
Research elective with the ability to present abstracts at national and international neurology and epilepsy conferences, including the AES and AAN

Research accomplishments

Our Pediatric Epilepsy Surgery Program boasts many research accomplishments since its inception in 2020 (Table 9). It ranks among the top pediatric neurology and neurosurgery programs nationally based on clinical outcomes, robust research contributions, fellowship training distinction, and innovative clinical practices. Substantial contributions have been made to national datasets [12-18]. Our program has also published extensively in the areas of epilepsy surgery outcomes, minimally invasive surgical techniques, long-term neuropsychological outcomes, and neuromodulation efficacy [18-21].

**Table 9:** Research Accomplishments PERC: Pediatric Epilepsy Research Consortium

Research Achievements
Ranks among the top pediatric neurology and neurosurgery programs nationally based on clinical outcomes, robust research contributions, fellowship training distinction, and innovative clinical practices
In 2023 and 2024, our Norton Children’s Hospital ranked in the top 50 nationally in neurology and neurosurgery on U.S. News & World Report’s list of Best Children’s Hospitals
Participates in multicenter studies, including the PERC and national epilepsy surgery registries
Contributions to national databases
Published extensively on epilepsy surgery outcomes, minimally invasive surgical techniques, long-term neuropsychological outcomes, and neuromodulation efficacy

## Discussion

Pediatric epilepsy centers across the US and worldwide

In 1990, the National Association of Epilepsy Centers (NAEC) established guidelines for services, personnel, and facilities in epilepsy centers [22] with revised guidelines in 2001 [23] and 2010 [24]. Four features encompass Level 3 and 4 epilepsy centers, including (1) interdisciplinary care team approach, (2) electrodiagnostic facilities, (3) safety protocols, and (4) patient education [24]. The primary goal was to attain complete seizure control, or a reduction in seizure frequency and/or medical side effects in patients with intractable epilepsy [24]. In 2020, the International League Against Epilepsy (ILAE) Pediatric Epilepsy Surgery Task Force established standardized criteria for pediatric epilepsy surgery center levels of care [5]. By analyzing 61 centers with experience in pediatric epilepsy surgery across 20 low-, middle-, and high-income countries, two levels of care were determined based on clinical complexities and institutional competencies. The levels of care were designed to improve safety and outcomes for pediatric epilepsy surgery and specify standards for personnel and technology. Our Pediatric Epilepsy Surgery Program qualifies for Level 4 care: across the pediatric age span; wide range of epilepsy etiologies; using advanced technologies such as 3T MRI, fMRI, PET, intra- or extra-operative invasive monitoring, and functional brain mapping; and establishing a multidisciplinary team skilled in pediatric neurology. Our program has met the standards of pediatric epilepsy surgery centers and ACNS guidelines for a pediatric epilepsy surgery clinic, surgical intervention, and EMU programs while offering cutting-edge technology.

Few articles in the literature have reported their own experiences with developing an epilepsy center in a low socioeconomic environment [25-30]. In Arifin and colleagues’ study of an epilepsy surgery program with limited resources in Indonesia, two periods were assessed: the first period when interictal EEG and MRI were used primarily for presurgical evaluation, while the second period used long-term non-invasive and invasive video-EEG [25]. These authors posited that stringent and selective criteria should be used to reserve surgeries for patients with well-understood pathophysiologies such as drug-resistant temporal lobe epilepsy [25]. Bäuerle et al.’s study of epilepsy surgery in low- and middle-income countries (LMIC) concurs with that of Arifin et al.’s, specifically, that new surgical epilepsy programs in LMIC should focus on patients with mesial temporal lobe epilepsy due to the favorable surgical outcome of eradicating seizures [25,30]. Furthermore, surgical epilepsy centers in LMIC should have a multidisciplinary team and use technologies and personnel that are available. Dugladze and colleagues describe their experience with establishing an epilepsy surgery program in the LMIC of Georgia [29]. Several features were highlighted, including training and mentoring for providers focusing on presurgical diagnosis and postoperative follow-up. In Jukkarwala and colleagues’ study of a low-cost epilepsy surgery center in India, identifying and operating on ideal epilepsy surgery candidates based on clinical history, video EEG data, and 1.5 T MRI were the key components [28]. The pediatric epilepsy surgical program in Vietnam is centered on the international collaboration of pediatric neurosurgeons, neurologists, and EEG technologists in the US and Vietnam [27]. Recognizing the neurosurgical health disparities among LMICs, the Duke Division of Global Neurosurgery and Neurology (DGNN) was established to emphasize service, research, and training [26]. The DGNN digitized health records and data systems and promoted research projects in epilepsy.

Our study in an underserved area coincides with the extant literature with respect to developing a Pediatric Epilepsy Surgery Program in an underserved region with limited resources. Most importantly is the establishment of a multidisciplinary team with each member receiving extensive and focused training for his/her particular position. Additionally, implementing an algorithm pathway from patient presentation to epilepsy surgery permits a structured technique for diagnosing patients and preparing them for potential surgical intervention. The main difference between our study of an underserved population compared to the literature on underserved areas is that ours focused on the development of an Epilepsy Surgery Program designed only for pediatric patients. In both Arifin et al.’s and Jukkarwala et al.’s studies, the patients were diagnosed with epilepsy as a child or adolescent, although their epilepsy surgeries were often performed at least 10 years later, when the patients were in their 20s. Our study featured pediatric patients who were not only diagnosed with epilepsy as children or adolescents but also underwent epilepsy surgery as pediatric patients.

Lessons learned

We have learned numerous lessons after establishing and expanding our Pediatric Epilepsy Surgery Program. We faced several challenges, including developing such a program in an underserved area where it was difficult to recruit and retain well-trained individuals (neurosurgeons with epilepsy surgery training, pediatric epileptologists, neuropsychologists, and experienced EEG technologists) to work in the center. After they were hired, each person needed to undergo extensive training to acquire the knowledge required for their specific role. Obtaining adequate finances to fund our program was another challenge. We contacted various philanthropic organizations and utilized our hospital’s financial resources to procure the specialized equipment required for pediatric epilepsy surgery. Physical space limitations were also a hurdle as we developed our program. Medical units at our hospital were rearranged as we designed and expanded our program. For example, the cardiology unit was moved to allow placement of the EMU. Additionally, by recognizing the limitations of our neurodiagnostic unit (limited EEG services, absence of continuous video EEG monitoring, and lack of specialized surgery assessments), we were subsequently able to significantly enhance and expand our neurodiagnostic competence by implementing infrastructure upgrades, staffing expansions, and innovative technologies.

Model for other Pediatric Epilepsy Surgery Programs

The development and growth of our Pediatric Epilepsy Surgery Program serves as a valuable model to other programs who are interested in starting and expanding a similar system. The most important aspect of our program is its multidisciplinary teamwork in both the inpatient and outpatient settings and between the myriad members of the program, including neurosurgeons, neurologists, nurses, administration, and housekeepers. We also stress the need for a visionary leader who is able to guide a new program from its infancy. Patience and tolerance are also beneficial attributes of team members. Our program is an excellent example of how an innovative and thriving center can be established in a relatively low socioeconomic city and state.

Future goals of our Pediatric Epilepsy Surgery Program

Future goals of our Pediatric Epilepsy Surgery Program include (1) expanding neuromodulation (DBS and RNS) programs, (2) implementing advanced diagnostic technologies (MEG and additional robotic assistance), (3) enlarging telemedicine and virtual care services to increase regional accessibility, (4) growing epilepsy fellowship and educational opportunities, and (5) augmenting involvement in national and international multicenter research projects.

Limitations

Several limitations within our study should be considered. Since our work was a single-center perspective of an underserved area, our account is represented by our healthcare environment, with its specific institutional support, philanthropic engagement, and access to tertiary care infrastructure. Certain aspects of our model, such as robotic-assisted sEEG or access to RNS, require substantial capital investment and institutional alignment, which may not be feasible for all programs. These factors may not be easily reproduced in all underserved environments, especially in settings with more limited health systems or minimal access to subspecialty care. We faced several challenges, such as recruitment of epilepsy-trained personnel, acquisition of advanced surgical technology, and education of referring providers, that were addressed over a multi-year period through advocacy and incremental growth. Other programs may have longer timelines or greater systemic barriers. Additionally, there was a lack of complete clinical outcome metrics (seizure freedom rates, neurodevelopmental outcomes, patient-reported quality of life, Engel classification of post-surgical seizure control, and patient/provider satisfaction data) in our study.

## Conclusions

The achievements of our Pediatric Epilepsy Surgery Program over a short period of time are due to many factors, including a (1) multidisciplinary collaboration among members of our center, (2) implementing advanced epilepsy diagnostic and surgical equipment and interventions, (3) expanding staffing who are adept in pediatric epilepsy techniques, (4) creating specialty epilepsy clinics, and (5) encouraging educational opportunities and research. These advancements have resulted in increased surgical volumes with greater complexity. Further expansion of the diagnostic, surgical, and neuromodulation capacities at our Pediatric Epilepsy Surgery Program will allow us to offer more innovative modalities to treat pediatric epilepsy.
